# Galectin-8 and -9 as prognostic factors for cervical cancer

**DOI:** 10.1007/s00404-022-06449-9

**Published:** 2022-04-04

**Authors:** Susanne Beyer, Maya Wehrmann, Sarah Meister, Theresa M. Kolben, Fabian Trillsch, Alexander Burges, Bastian Czogalla, Elisa Schmoeckel, Sven Mahner, Udo Jeschke, Thomas Kolben

**Affiliations:** 1 Department of Obstetrics and Gynecology, University Hospital, LMU Munich, Marchioninistr. 15, 81377 Munich, Germany; 2 Institute of Pathology, University Hospital, LMU Munich, Marchioninistr. 15, 81377 Munich, Germany; 3 Department of Obstetrics and Gynecology, University Hospital, Universitätsklinikum Augsburg, Stenglinstr. 2, 86156 Augsburg, Germany

**Keywords:** Galectin-8, Galectin-9, Cervical cancer, Survival

## Abstract

**Purpose:**

Galectins are carbohydrate-binding proteins with multiple effects on cell biology. Research shows that they play an important role in tumor development and progression. Therefore, in this study, the presence of Galectin-8 and -9 (Gal), both already known as prognostic factors in other tumor entities, were investigated in cervical cancer. Our aim was to examine the association of Gal-8 and -9 expression with histopathological markers and survival of the patients.

**Methods:**

Gal-8 and -9 expression was investigated in 250 cervical cancer samples by immunohistochemistry. The staining was evaluated using the immunoreactive score (IRS). The results were correlated to clinical and pathological data. The correlation of Gal-8 and -9 expression with overall and relapse-free survival was analyzed.

**Results:**

Expression of Gal-8 was associated with negative N-status and lower FIGO status. Detection of Gal-9 was connected to negative N-status and lower grading regarding all specimens. A correlation of Gal-9 with lower FIGO status was detected for squamous cell carcinoma (SCC) only. Expression of Gal-8 was associated with relapse-free survival of SCC patients in a positive manner. Gal-9 expression was associated with better overall survival.

**Conclusion:**

Our results suggest that expression of both galectins is inversely associated with tumor stage and progression. Gal-8 expression is associated with relapse-free survival of patients with SCC, while presence of Gal-9 in cervical cancer is associated with a better prognosis in regard of overall survival.

**Supplementary Information:**

The online version contains supplementary material available at 10.1007/s00404-022-06449-9.

## Introduction

Cervical cancer is the fourth most common cancer and the fourth leading cause of cancer death among women worldwide. In 2018, 570,000 new cases of cervical cancer were registered and 311,000 women died due to it [1].

A persistent infection with high-risk HPV types is the leading factor for the development of cervical cancer, but there are several other components, which can cause disease progression, like immunosuppression, genetic predisposition or smoking [2–5].

Although, the incidence and mortality of cervical cancer were decreased in Germany in the last decades by the introduction of a screening program, cervical cancer remains a global major health problem. In contrast to many other cancer types, cervical cancer survival rates have not improved since the 1970s, which reflects a lack of treatment [6, 7]. Especially for patients with relapse or metastatic disease, therapeutic options are limited. For that reason, the development of further prognostic factors and therapeutic targets is necessary.

In the last decades, galectins have attracted interest in cancer research. Not only because of their broad spectrum of functions, but also because of their potential as prognostic factors and new targets in cancer therapy [8, 9].

Galectins are proteins with a highly conserved sequence of 130 amino acids and a ß-galactoside-binding site, the carbohydrate recognition domain (CRD) [10]. These proteins have the ability to interact with other non-galactosylated binding partners through the CRD or other parts of the galectin [11]. Depending on their molecular structure, galectins are classified into three subgroups: the “prototype” galectins (Gal-1, Gal-2, Gal-5, Gal-7, Gal-10, Gal-11, Gal-13, Gal-14 and Gal-15), which have one CRD and often dimerize, the “tandem-repeat” galectins (Gal-4, Gal-6, Gal-8, Gal-9 and Gal-12) with two CRDs connected by a linker peptide and the chimeric Gal-3 with one C-terminal CRD and a nonlectin N-terminal domain [8, 12]. Galectins can be located cytosolic, in the nucleus, at membranes and in the extracellular matrix [13, 14]. As they lack a signal peptide, galectins are secreted extracellular by an atypical secretory mechanism [15]. They bind glycans at the cell surface or extracellular matrix, but also non-carbohydrate ligands in the cytosol and nucleus. Because of their wide-ranging binding-capacities, they have multiple effects on cell biology: Galectins are implicated in cell–cell-, cell–matrix–interaction, the modulation of intracellular signaling and cellular functions [14, 16].

Many studies in the last years have shown that tumor cells present an increased expression of galectins and that these proteins play an important role in the promotion and progression of a tumor. They are known to affect, for example, proliferation signals, angiogenesis, tumor invasion and metastasis, immune escape and cell death resistance. Depending on the type of cancer, galectins may act as tumor-promotors or tumor-suppressors [17]. Therefore, galectin expression is frequently usable as a prognostic marker for a cancer patients’ survival [18–20].

In this study, we are focusing on two tandem-repeat galectins, Galectin-8 and -9, both known to be associated to malignancies, among them other gynecological malignancies like ovarian cancer [21].

Galectin-8 (Gal-8) is a lectin. It is well known to modulate cell adhesion and angiogenesis in tumor cells and has been evaluated as a prognostic factor for different cancer types such as multiple myeloma, ovarian, gastric and urothelial cancer by immunohistochemistry, PCR and Western blot [21–24]. Galectin-9 (Gal-9) has been described as a protein with many roles in tumor development and as a prognostic factor in different entities of cancer. In many other solid tumors, higher Gal-9 expression has been associated with lower tumor progression and better overall and progression-free survival [21, 25, 26].

A few studies have investigated the role of galectins in cervical cancer, but to our knowledge, none of these has examined the role of Gal-8 and only few studies analyzed the role of Gal-9 [27, 28]. In 2008, Liang et al. investigated immunohistochemically the Gal-9 expression in cervical intraepithelial neoplasia (CIN; 17 patients), squamous cell carcinoma (SCC; 38 patients) and normal squamous cell epithelium of the cervix in a rather small panel. In that study, decreased Gal-9 expression was associated to the malignant potential in SCC [27]. In summary, the number of studies on Gal-8 and -9 in cervical cancer is limited and their prognostic value in cervical cancer still needs to be elucidated.

Therefore, the aim of this study was to evaluate the expression of Gal-8 and -9 in cervical cancer using immunohistochemistry and to analyze their correlation to histopathological and clinical markers in cervical cancer.

## Materials and methods

### Patients and specimens

250 Paraffin-embedded cervical cancer samples were acquired from patients who underwent surgery during 1993–2002 in the Department of Obstetrics and Gynecology of the Ludwig-Maximilians-University of Munich. The median age of the patients in the collective was 47.0 years, with a median overall survival of 100 months. The collective contained patients with squamous cell carcinoma, adenocarcinoma and adenosquamous carcinoma. Other histological subtypes were excluded due to low number.

The distribution of histopathological markers is listed in Table 1. For positive controls of the immunohistochemical staining, we utilized colon tissue for Gal-9 and placenta tissue for Gal-8, both received from the Department of Obstetrics and Gynecology of the Ludwig-Maximilians-University of Munich. Clinical and follow-up data were provided by the Munich cancer registry (request from April 15th, 2020) and retrieved from medical records.

**Table 1:** Distribution of histopathological parameters

Item	No./total no.	%
Age (in years)		
< 49	121/244	49.6
≥ 49	123/244	50.4
Histological subtype		
Sqaumous	194/244	77.5
Adenocarcinoma	34/244	13.9
Adenosquamous	15/244	6.1
Not available	1/244	0.4
Tumor size, pT		
pT1	107/244	43.9
pT2	126/244	51.6
pT3	9/244	3.7
pT4	1/244	0.4
Not available	1/244	0.4
Lymph-node status		
N −	145/244	59.4
N +	98/244	40.2
Not available	1/244	0.4
FIGO		
I	79/244	32.4
II	64/244	26.2
III	93/244	38.1
IV	7/244	2.9
Not available	1/244	0.4
Tumor grade		
I	21/244	8.6
II	143/244	58.6
III	78/244	32.0
Not available	2/244	0.8
Progression (over 235 months)		
None	180/244	73.8
At least one	63/244	25.8
Not available	1/244	0.4
Survival (over 235 months)		
Right censured	211/244	86.5
Died	33/244	13.5

### Ethics approval

All cervical cancer specimens were collected for histopathological diagnostics. Clinical tests were completed, when they were recruited for this study. Patient data were fully anonymized and the authors were blinded for any information during experimental analyses. The study was conducted conforming to the Declaration of Helsinki and was approved by the local ethics committee of the Ludwig-Maximilians-University of Munich (reference number 259-16, 2016).

### Immunohistochemistry

Paraffin-embedded and formalin-fixed samples got processed to tissue microarrays (TMAs) in the pathological institute of the Ludwig-Maximilians-University of Munich. The slides were first deparaffinized in Roticlear, a xylol-replacement medium. After washing the tissue in 100% ethanol, the endogenous peroxidase was blocked with 3% methanol/hydrogen peroxide. The specimens were then rehydrated in a descending series of alcohol (100%, 75%, 50%). Heat induced antigen-retrieval was performed by cooking the sections in trisodium citrate buffer (pH = 6) in a pressure cooker at 100 °C for 5 min. After rinsing the slides in distilled water and PBS-buffer, a blocking solution was added to obviate unspecific hydrophobic bindings between immunoglobulins and fatty tissue or cell membranes. The samples were incubated for 16 h at a temperature of 4 °C with the primary antibodies afterward (Table 2). By applying a post-block-reagent and the HRP-polymer, the staining was intensified. The substrate-staining with DAB was performed and followed by the counterstaining with Mayer acidic hematoxylin. The specimens were dehydrogenated in a rising alcohol series (70%, 96% and 100%) and covered. Detailed information about the suitable detection system and precise steps are specified in Table 2.

**Table 2:** Staining procedure

Anti galectin-8^1^	Anti galectin-9^2^
Blocking solution^3^: 5 min	Blocking solution^3^: 5 min
Primary antibody^1^: 1:150 in PBS^4^	Primary antibody^2^: 1:300 in PBS^4^
Incubation 1 h at room temperature	Incubation 16 h, 4 °C
PostBlock^3^: 20 min	PostBlock^3^: 20 min
HRP Polymer^3^: 30 min	HRP Polymer^3^: 30 min
Chromogen: DAB^5^ (1 min)	Chromogen: DAB^5^ (1 min)

^1^Anti galectin-8 (rabbit IgG monocolonal, clone EPR4857), concentration not determined, company: Abcam (Cambridge, UK), order number: ab109519

^2^Anti galectin-9 (rabbit IgG polyclonal), concentration: 1 mg/mL, company: Abcam, order number: ab69630

^3^ZytoChem Plus HRP Polymer Kit (Mouse/Rabbit) 3 × 100; company: Zytomed Systems (Berlin Germany)

^4^Dulbeccos’ Phosphate Buffered Saline

^5^Liquid DAB + Substrate Chromogen System 1 mg/mL, DAKO

The extent of the expression was evaluated by the immunoreactive score (IRS). This semiquantitative score consists of two scales that measure the intensity of the staining (0 = not stained, 1 = low intensity, 2 = medium intensity, 3 = high intensity) and the percentage of the stained tumor cells (0 = 0%, 1 = 1–10%, 2 = 11–50%, 3 = 51–80%, 4 ≥  80%). Finally, both scales are multiplied, the IRS has a range from 0 = no expression to 12 = very high expression.

### Statistical analysis

IBM SPSS Statistics version 26 (Armonk, NY, USA) was used to perform statistical analysis. Bivariate correlations were calculated by Spearman’s-rank-correlation coefficient and non-parametric tests (NPAR: Mann–Whitney-*U* test, Kruskal–Wallis test) were performed to compare independent groups. Kaplan–Meier-curves and log-rank-test (Mantel-Cox) were used for survival analysis. Survival times are shown in months. *p* had to be < 0.05 to show a significant statistical difference.

## Results

### Galectin-8 staining in cervical cancer

As a positive control, we used non-pathological colon tissue that showed a strong cytoplasmatic expression in > 90% of the epithelial cells (Supplement 1).

A total of 77.9% of the samples were stained with Gal-8, with a median Immune Reactive Score (IRS) of 4 (16.4%), while 9.4% of the spots did not express Gal-8 (IRS = 0). 12.7% of the samples could not be evaluated due to missing tumorous tissue. 58.2% of the evaluated spots had an enhanced expression (IRS ≥ 4). Gal-8 was only expressed in the cytoplasm, there was no nuclear staining.

We further investigated the correlation between Gal-8 and different histopathological markers like histological subtype, T-status, N-status, grading and FIGO-status. For the distribution of these parameters in our collective, see Table 1.

**Fig. 1:**
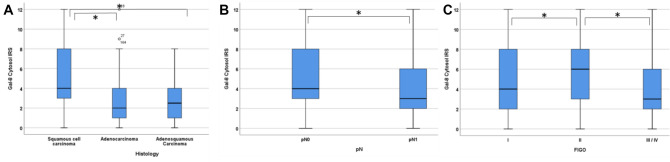
Gal-8 staining results. Significant differences regarding the histological subtype and Gal-8 expression with a significant higher Gal-8 expression in squamous cell carcinomas (**A**). Significant higher Gal-8 expression in patients with lymph-node negativ status (**B**). Patients with FIGO II showed an enhanced Gal-8 expression compared to FIGO I and FIGO III/IV (**C**). Significant differences are marked by “*”

A statistically significant difference between the expression of Gal-8 in the three histological subtypes could be found (*p* = 0.024; *ρ* = − 0.208 with *p* = 0.002; Table 3), with a median IRS of 4 for the squamous cell carcinomas, compared to a median IRS of 2 in the group of the adenocarcinomas. In adenosquamous carcinomas, the median IRS was 2.5 (Fig. 1A; Supplement 2).

**Table 3:** Correlation to histopathological markers

	Galectin-8				Galectin-9			
	Median IRS (± SD)	%	*p* (NPAR)	*ρ* (Rho)	Median IRS (± SD)	%	*p* (NPAR)	*ρ* (Rho)
Histology			**0.024**	− 0.208 (*p* = 0.002)			0.588	–
SCC	4 (± 3.23)	18.3			2 (± 2.39)	20,8		
Adeno-Ca	2 (± 3.28)	20.7			4 (± 2.77)	31		
Adenosquamous Ca	2.5 (± 2.50)	N.a			2 (± 2.71)	15,4		
Others	4 (± 0.00)	100.0			3 (± 0.00)	100		
pT			0.687				0.36	–
T1	4 (± 3.24)	19.4			3 (± 2.43)	17,8		
T2	4 (± 3.10)	18.6			2 (± 2.44)	22,1		
T3	2.5 (± 4.92)	N.a			0,5 (± 3.25)	N.a		
T4	2 (± 0.00)	100			6 (± 0.00)	100		
pN			**0.004**	− 0.277 (*p* < 0.001)			**0.024**	− 0.157 (*p* = 0.023)
N −	4 (± 3.28)	16.5			3 (± 2.27)	17.7		
N +	3 (± 3.03)	10.6			2 (± 2.68)	18.6		
FIGO			**0.008**	− 0.165 (*p* = 0.016)			0.067	–
FIGO I	4 (± 3.45)	16.2			3 (± 2.41)	15.2		
FIGO II	6 (± 3.06)	8.5			3 (± 2.13)	21.1		
FIGO III/IV	3 (± 3.03)	10.5			2 (± 2.67)	18.4		
FIGO (SCC only)	–	–	–				**0.002**	− 0.247 (*p* = 0.001)
FIGO I	–	–	–		3 (± 2.30)	19.6		
FIGO II	–	–	–		3 (± 2.46)	26.7		
FIGO III/IV	–	–	–		1 (± 2.40)	19.7		
Grading			0.865				**0.048**	− 0.159 (*p* = 0.040)
G1	4 (± 3.79)	5.3		–	4 (± 2.59)	22.2		
G2	4 (± 3.18)	19.7			2 (± 2.26)	23.1		
G3	4 (± 3.24)	20.0			2 (± 2.69)	15.5		

Significant results are shown in bold

*N.a.* not available

Regarding the N-status, patients with lymph-node negative status (N −) showed a median IRS of 4, while the group with lymph-node positive (N +) status had a median IRS of 3. So Gal-8 expression correlated negatively with the N-status, meaning that higher Gal-8 expression was associated with a negative N-status (Fig. 1B; Supplement 2; *p* = 0.004, *ρ* = − 0.277 with *p* < 0.001; Table 3).

We detected a statistically significant correlation between lower FIGO status and higher Gal-8 expression (*p* = 0.008; *ρ* = − 0.165 with *p* = 0.016, Fig. 1C and Table 3). Patients with FIGO I had a median IRS of 4, represented by 16.2% of the samples, compared to a median IRS of 6 in patients with FIGO stage II (8.5%). Higher FIGO stages (III and IV) had an IRS of 3 (Supplement 2).

Altogether, Gal-8 was associated with histological subtype (*p* = 0.003), negative N-status (*p* = 0.011) and low FIGO-status (*p* = 0.016). Regarding the grading (*p* = 0.865) or T-status (*p* = 0.687), no significant difference could be found.

### Galectin-9 staining in cervical cancer

To control the quality of the Gal-9 staining, we used normal placenta tissue, where a cytoplasmic expression could be found in all trophoblastic cells (Supplement 1).

Regarding cervical carcinoma, all samples showed only a cytoplasmic expression of Gal-9. 69.3% of the specimens were stained, while 17.2% had an IRS of 0 and 13.5% could not be evaluated due to missing tumor tissue. 16.8% of the evaluated samples presented the median IRS of 2.

**Fig. 2:**
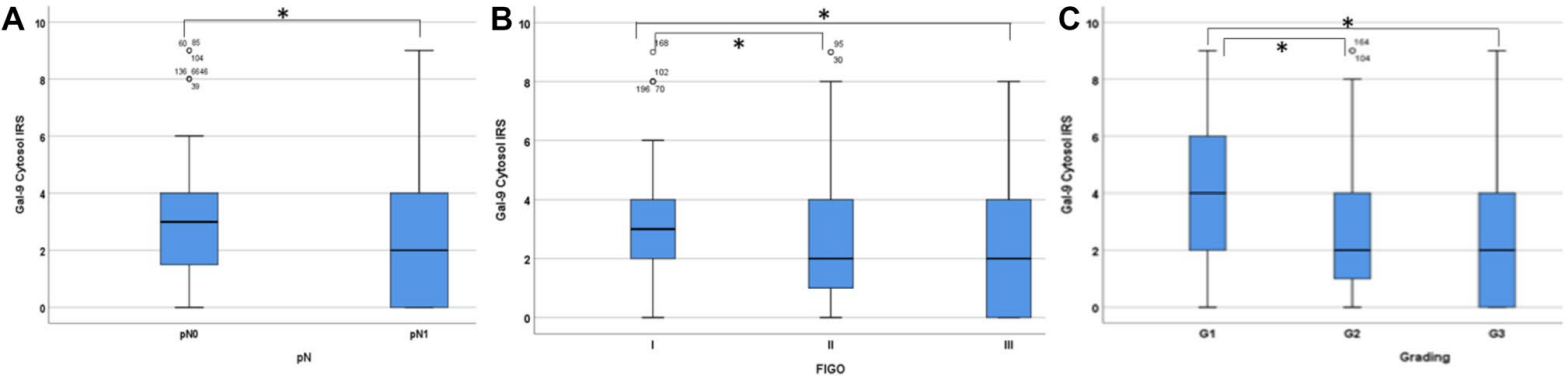
Gal-9 staining results. Significant higher Gal-9 expression in patients with lymph-node negativ status (**A**). Patients with FIGO stage I and SCC presented higher Gal-9 expression than patients with FIGO stage III or IV (**B**). Significant enhanced expression of Gal-9 in low graded tumors (**C**). Significant differences are marked by “* ”

The investigation of the lymph-node status of the patients showed that negative N-status was correlated to altered Gal-9 expression (Fig. 2A; *p* = 0.024; *ρ* = − 0.157 with *p* = 0.023, Table 3). Patients with no lymph-node metastasis presented a median IRS of 3, with 17.7% samples showing this IRS. In contrast, 18.6% of the specimens with a positive N-status showed a median IRS of 2 (Supplement 3).

There was no significant difference between FIGO stage and Gal-9 in general (*p* = 0.067; Table 3). However, regarding only the subgroup of SCC, Gal-9 expression was correlated to FIGO stages. In FIGO I and SCC, the median IRS was 3, while in FIGO II and SCC, the median IRS was 2, as well as in FIGO III/IV patients.

Thus, we could show a statistically significant correlation between higher Gal-9 expression and lower FIGO stage in SCC (*p* = 0.002; *ρ* = − 0.247 with *p* = 0.001, Table 3; Fig. 2B; Supplement 3).

Furthermore, a statistically significant association between higher Gal-9 expression and lower grading could be shown. Low graded tumors (G1) did not represent the general median IRS of 2 but showed a median IRS of 4 in 22.2% of the cases. Specimens with intermediate grading (G2) had a median IRS of 2 in 23.1% of the samples. For the high graded (G3) samples, the median IRS was also 2, shown by 15.5% of the cases. Therefore, enhanced Gal-9 staining was associated with lower grading (*p* = 0.048; Rho = − 0.159 with *p* = 0.040; Table 3; Fig. 2C; Supplement 3).

Altogether, we could find correlations of cytoplasmic Gal-9 expression and N-status (*p* = 0.024), FIGO stage in SCC (*p* = 0.002) and for grading (*p* = 0.048). No statistically significant association could be detected for histological subtype or T-status.

### Role of Galectin-8 and -9 for overall survival

**Fig. 3:**
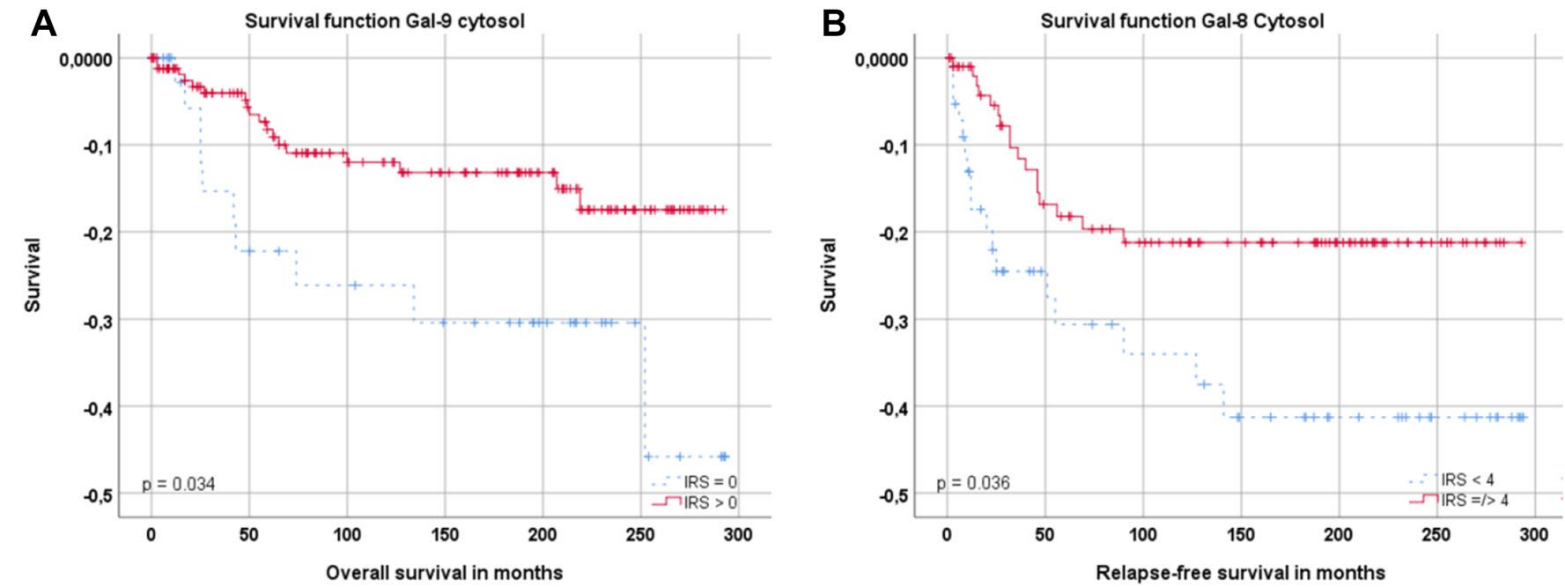
**A** Kaplan–Meier analyses for overall survival: Galectin-9 (*p* = 0.034, **A**) with cytoplasmic expression compared (IRS ≥ 1) to no cytoplasmic expression (IRS = 0). **B** Kaplan–Meier analyses for relapse-free survival: low cytoplasmic Galectin-8 expression (IRS < 4) compared to higher Galectin-8 expression (IRS ≥ 4) regarding RFS in patients with SCC (*p* = 0.036)

An analysis on overall survival showed that expression of Gal-9, but not Gal-8 was associated with better overall survival (OS): high expression of Gal-9 (IRS ≥ 1) was correlated with better prognosis in overall survival of the patients (*p* = 0.034). For the Kaplan–Meier curve, see Fig. 3A. This coherence matches with our described findings concerning N-status, FIGO stage and grading.

### Role of Galectin-8 and -9 for relapse-free survival

In addition to the overall survival, we also investigated the role of Gal-8 and -9 for relapse-free survival (RFS). There, we could find that the expression of Galectin-8 was positive correlated to RFS.

Higher Gal-8 expression (IRS ≥ 4) was significantly positive correlated to the relapse-free survival, but only in the histological subgroup of squamous cell carcinomas (*p* = 0.036, Fig. 3B).

This result matches with the influence of the galectins on the histopathological markers, meaning that elevated Gal-8 expression was associated with negative N-status, low FIGO-stage and better RFS-rates.

### Cox regression of Galectin-8 and -9 and clinical pathological variables

The additionally performed multivariate cox-regression tested which histopathological parameters were independent prognosticators for survival in our study-group.

For overall survival, the histological subtype (*p* = 0.004) and the T-stage (*p* = 0.023) were independent prognosticators, but not Gal-8-, Gal-9-expression or other tested clinic pathological parameters (Table 4).

**Table 4:** Cox-regression for overall survival

Variable	Significance	Hazard ratio of Exp(*B*)	Lower 95% CI of Exp(*B*)	Upper 95% CI of Exp(*B*)
Age	0.666	0.992	0.959	1.027
Histology	**0.004**	2.106	1.271	3.489
pT	**0.023**	2.847	1.154	7.026
pN	0.889	0.890	0.175	4.530
Grading	0.923	0.992	0.884	1.166
FIGO	0.415	1.775	0.446	7.058
Galectin-8 cytoplasm	0.528	1.045	0.911	1.199
Galectin-9 cytoplasm	0.410	0.930	0.783	1.105

Significant results are shown in bold

Regarding the relapse-free survival, the histological subtype (*p* = 0.028) turned out to be an independent prognosticator, but neither Gal-8 or -9 expression, nor the other tested parameters were independent prognostic factors (Table 5).

**Table 5:** Cox-regression for relapse-free survival

Variable	Significance	Hazard ratio of Exp(*B*)	Lower 95% CI of Exp(*B*)	Upper 95% CI of Exp(*B*)
Age	0.835	1.003	0.978	1.207
Histology	**0.028**	1.565	1.049	2.336
pT	0.147	1.598	0.848	3.010
pN	0.695	1.304	0.345	4.925
Grading	0.837	0.990	0.901	1.088
FIGO	0.379	1.573	0.574	4.316
Galectin-8 cytoplasm	0.926	1.005	0.909	1.111
Galectin-9 cytoplasm	0.741	0.980	0.869	1.105

Significant results are shown in bold

## Discussion

In this study, we examined Galectin-8 and -9 in cervical cancer samples.

By immunohistochemical analysis, we detected an association of Gal-8 with histological subtype, negative N-status and low FIGO stage. Gal-9 expression correlated with negative lymph-node status, low grading and—in SCC—with lower FIGO stages. While Gal-9 expression is associated to overall survival rates, Gal-8 expression is correlated with better relapse-free survival in patients with squamous cell carcinoma.

With their effect on the cell cycle, the family of galectins plays an important role in the tumor biology. By different pathways, they can influence proliferation, apoptosis, metastasis, angiogenesis and even immune response.

Gal-8 is a protein that was cloned in 1995 from rat liver cDNA expression library [29]. The human Gal-8 gene encodes for seven different isoforms [30, 31]. Several studies have shown the important role of Gal-8 as a modulator of tumor development and progression: in non-small cell lung carcinoma, Gal-8 attenuates cell-adhesion and induces the apoptotic process through interactions with integrins [32]. Regarding endothelial cells, it binds to the transmembranous glycoprotein CD166 that may mediate pro-angiogenic and -migratory effects [33]. In colon-cancer cells, a higher cytosolic Gal-8 expression lead to reduced migration and growth rate [34]. Our results suggest a correlation of high Gal-8 expression with low tumor stages and better progression-free survival, which matches the already described pro-apoptotic effects of Gal-8 and its effects in colorectal cancer. To our knowledge, this is the first study regarding Gal-8 expression in cervical cancer in general. Based on these results, further studies are needed to clarify its specific impact on tumor biology and to precise the influence of its different isotypes, which is technically not possible by immunohistochemistry so far.

Since it was cloned from Hodgkin-Lymphoma in 1997 [35], multiple functions in tumor biology have been reported for Gal-9. In addition to its pro-apoptotic and anti-metastatic potential, Gal-9 acts as an important modulator of immunity and inflammation and is known as an eosinophil chemoattractant [26, 36, 37]. Loss of Gal-9 in cancer cells is frequently correlated with tumor progression [38]. In breast cancer, Gal-9 was shown to be involved in cell aggregation, preventing metastasis [39]. Gal-9 was described as a potent inducer of apoptosis, for example, by caspase-dependent or mitochondria mediated pathways in lymphoma cell-lines [40] or ovarian cancer, respectively. Thereby, it suppresses tumor growth [41, 42]. Similarly to Gal-8, alternative splicing and proteolytic processing of the Gal-9 gene (LGALS9) leads to multiple Gal-9 isoforms. It has been found that endothelial cells express five of these splicing variants and that Gal-9 also plays a role in angiogenesis, as its expression is increased in activated and tumor endothelial cells [43].

Additionally, Gal-9 protects the tumor cells against cytotoxic cell-dependent killing [44]. In summary, Gal-9 presents an immunosuppressive activity, which may enable tumor immune escape, which suggests a poor prognosis for cancer patients with enhanced Gal-9 expression. In contrast, the function in metastasis, apoptosis and cell adhesion implicates a positive effect on the outcome of tumors with higher Gal-9 expression. Our results go along with these observations as high Gal-9 expression was correlated to lower tumor stages and better overall-survival rates. A significant correlation between Gal-9 expression and overall-survival is also verified at the level of gen-expression (LGALS9) [45]. As well as with Gal-8, it is not possible to distinguish the different isoforms of Gal-9. Examining these isoforms separately could help to understand the immunosuppressive effect on the one hand and the pro-apoptotic-effect on the other hand.

We detected Gal-8 as well as Gal-9 only in the cytoplasm but not in the nucleus. Little data exist respecting Gal-8 location for survival analysis: in breast cancer, nuclear Gal-8 expression did not show any significant relevance regarding survival rates, while cytoplasmic Gal-8 expression correlated to better survival rates [46]. Not only in our study, also in other cancer types, Gal-8 was also not detectable in the nucleus [34, 47]. A missing nuclear Gal-9 expression in cervical cancer was also described in ovarian cancer and breast cancer [21, 39]. To the best of our knowledge, there are no data analyzing the influence of the location of Gal-9 expression on its ongogenic or tumorsuppressive effects. For both galectins, Gal-8 and Gal-9 the impact of their location may be an interesting aspect for further studies.

This is the first study, which examined the correlation of Gal-8 and -9 to histopathological markers in a representative sample of cervical cancer. As adenosquamous and adeno-carcinomas are rare histological subtypes, their number included in our study was also limited which has to be noted. Whether or how the different isoforms of Gal-9 may influence tumor biology and the patient’s outcome remains to be elucidated in further studies.

## Conclusion

In our study, Galectin-8 and -9 expression were examined in 250 cases of cervical cancer. We showed that expression of both galectins is inversely associated with FIGO stage and progression. Gal-8 expression is a positive prognostic factor for relapse-free survival of patients with SCC, while presence of Gal-9 in cervical cancer is correlated to a better prognosis regarding overall survival.

## Supplementary Information

Below is the link to the electronic supplementary material.

Supplementary file1 (DOCX 9424 KB)

## Abbreviations

CIN: Cervical intraepithelial neoplasia
CRD: Carbohydrate recognition domain
IRS: Immunoreactive Score
N.a.: Not available
OS: Overall survival
RFS: Relapse-free survival
SCC: Squamous cell carcinoma
