# Supplementary material for: Galectin-8 and -9 as prognostic factors for cervical cancer
Source: Arch Gynecol Obstet. 2022 Apr 4;306(4):1211–20. doi: 10.1007/s00404-022-06449-9 (PMC9470666; doi:10.1007/s00404-022-06449-9)
Supplement: Supplementary file 1 — Supplementary file1 (DOCX 9424 KB) [file 404_2022_6449_MOESM1_ESM.docx]

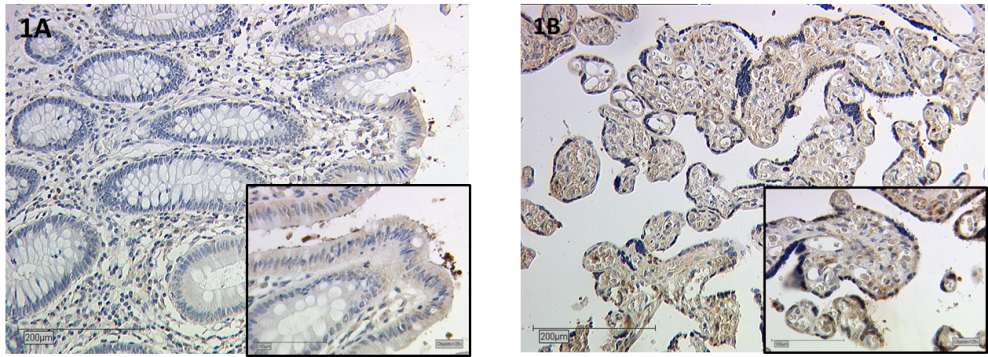


**Supplement 1**. Positive control of Gal-8 staining in colon tissue with strong cytoplasmic expression in epithelial cells (**A**). Positive Control of Gal-9 staining in placenta tissue with strong cytoplasmic expression in trophoblastic cells (**B**).


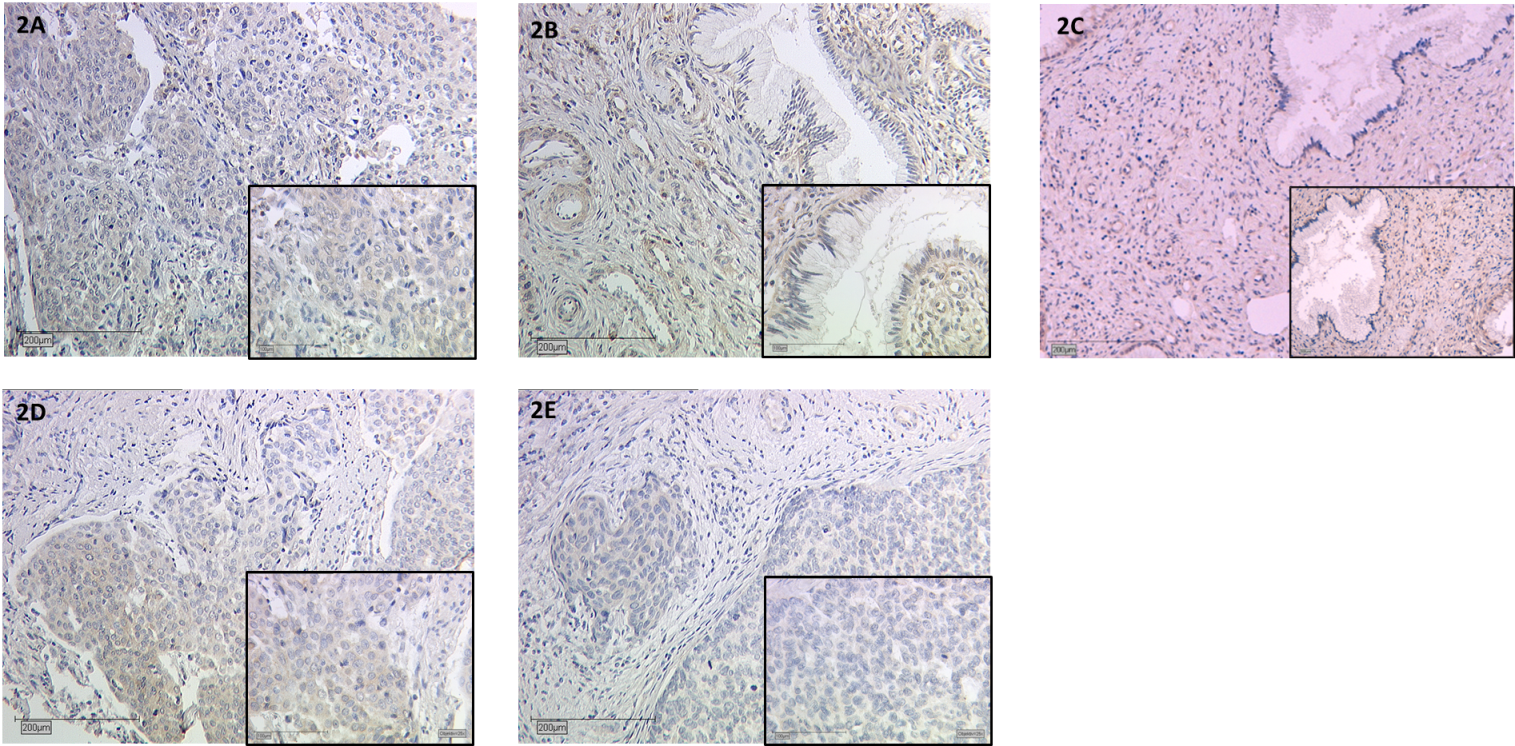


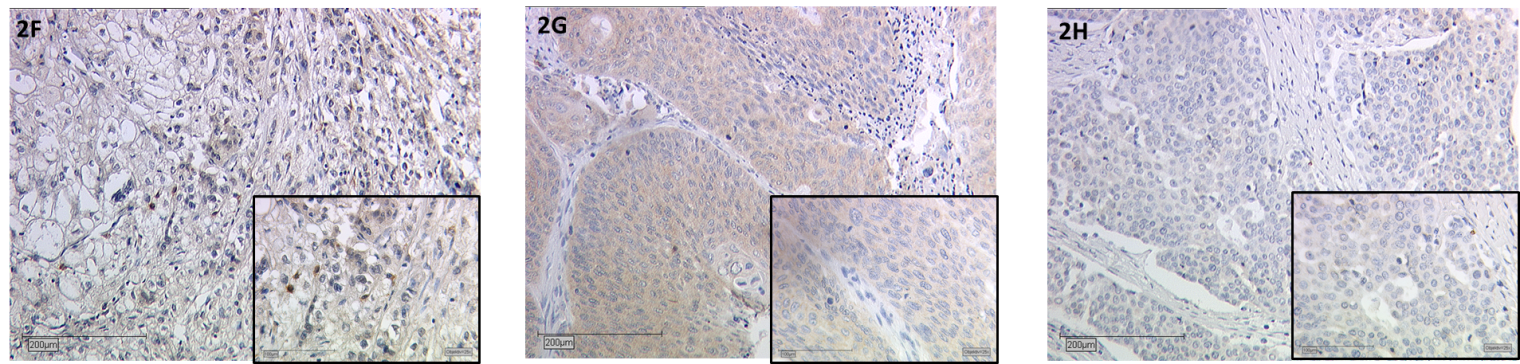


**Supplement 2:** Gal-8 staining. Squamous cell carcinoma (**A**) showed a median expression of Gal-8, while adenocarcinomas showed a signficant lower expression (**B**) as well as adenosquamous carcinomas (**C**); N-Status: patients with lymph-node negative status showed a median expression of Gal-8 (**D**). Patients with lymph-node positive status showed a significant lower expression (**E**). FIGO: patients with FIGO I had a median expression of Gal-8 (**F**), while patients with FIGO II showed enhanced expression (**G**) and patients with FIGO III or IV had a significant lower expression (**H**)**.**


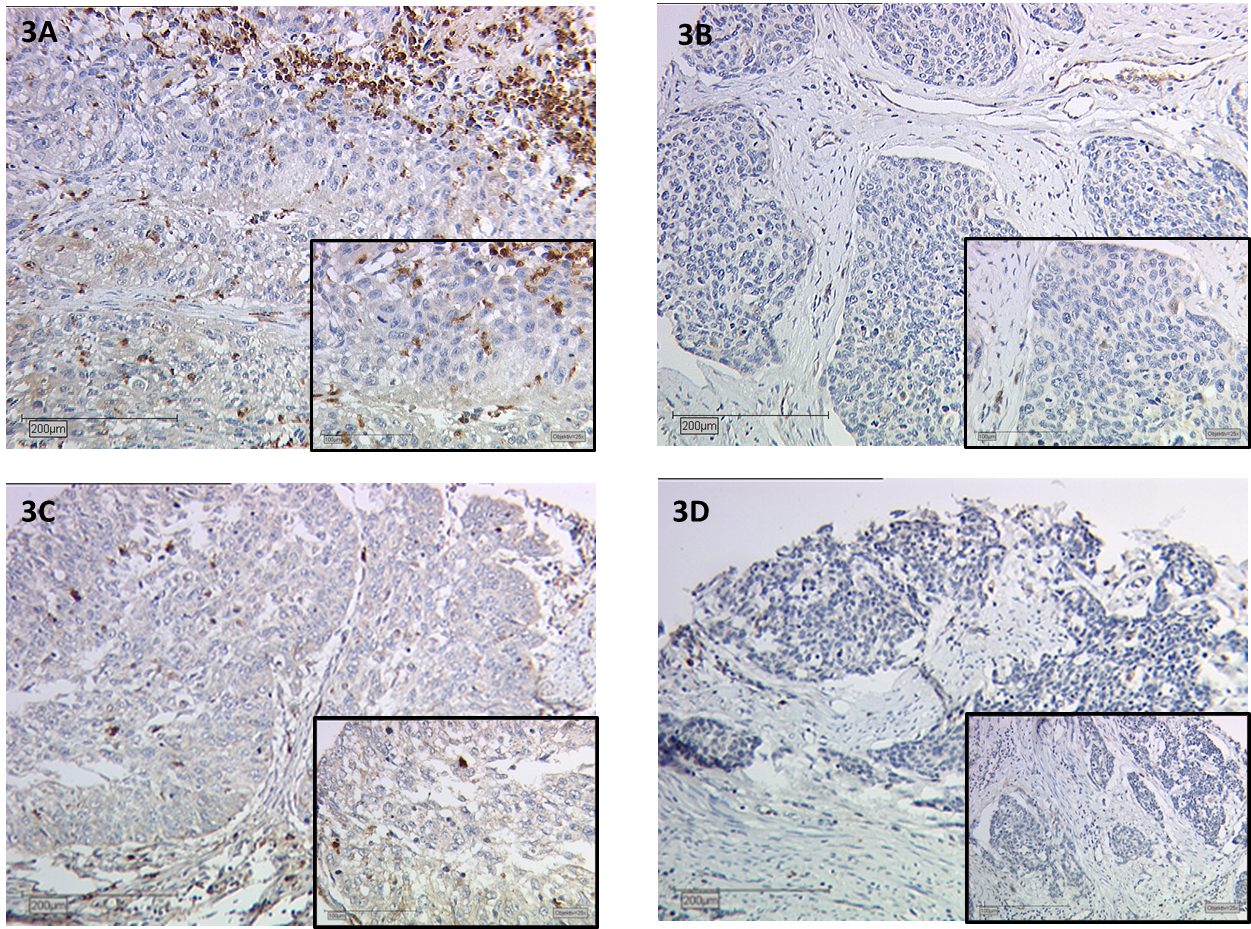


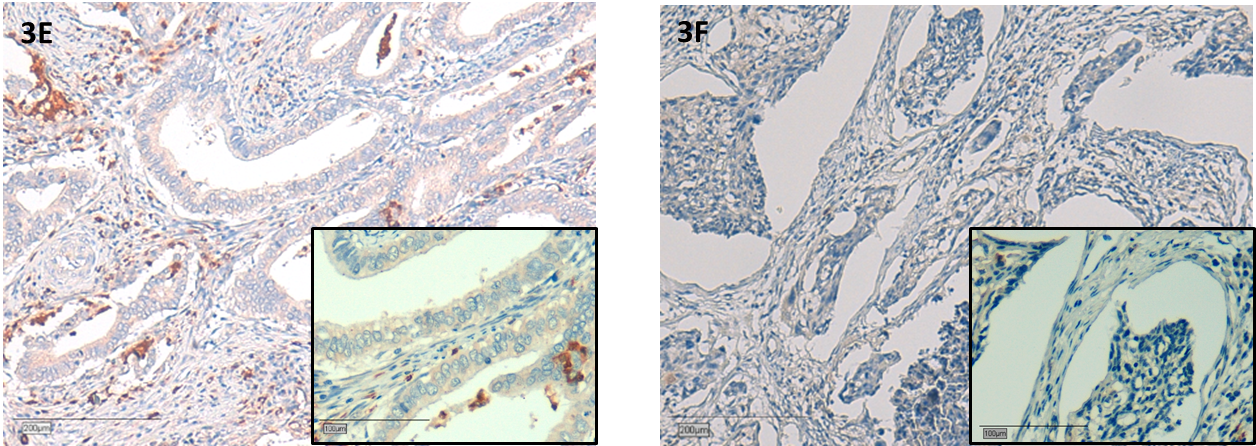


**Supplement 3:** Gal-9 staining. Tumors with no lymph-node metastasis showed significantly higher expression (**A**) than tumors with lymph-node positive status (**B**); Patients with FIGO stage I and SCC presented higher Gal-9 expression (**C**) than patients with FIGO stage III or IV (**D**). Low graded (G1) tumors showed enhanced expression of Gal-9 (**E**), compared to higher graded tumors (G3), which showed decreased expression (**F**).
